# Stereo Vision: The Haves and Have-Nots

**DOI:** 10.1177/2041669515593028

**Published:** 2015-07-30

**Authors:** Robert F. Hess, Long To, Jiawei Zhou, Guangyu Wang, Jeremy R. Cooperstock

**Affiliations:** McGill Vision Research, Department of Ophthalmology, McGill University, Montreal, Canada; Centre for Intelligent Machines, McGill University, Montreal, Canada; McGill Vision Research, Department of Ophthalmology, McGill University, Montreal, Canada; Centre for Intelligent Machines, McGill University, Montreal, Canada; Centre for Intelligent Machines, McGill University, Montreal, Canada

**Keywords:** Stereopsis, General population, have-nots

## Abstract

Animals with front facing eyes benefit from a substantial overlap in the visual fields of each eye, and devote specialized brain processes to using the horizontal spatial disparities produced as a result of viewing the same object with two laterally placed eyes, to derived depth or 3-D stereo information. This provides the advantage to break the camouflage of objects in front of similarly textured background and improves hand eye coordination for grasping objects close at hand. It is widely thought that about 5% of the population have a lazy eye and lack stereo vision, so it is often supposed that most of the population (95%) have good stereo abilities. We show that this is not the case; 68% have good to excellent stereo (the haves) and 32% have moderate to poor stereo (the have-nots). Why so many people lack good 3-D stereo vision is unclear but it is likely to be neural and reversible.

## Introduction

Much is known about the properties of stereo processing (Howard & Rogers, 2002), what computations are involved (Barlow, Blakemore, & Pettigrew, 1967) and where in the brain stereo-processing occurs (Neri, 2004). Even though 3-D movies, 3-D television technology, and 3-D apps for mobile devices are commonplace, the percentage of the general population that possesses normal stereopsis is a matter of controversy. Although it is commonly believed to be about 95% (Birch et al., 2008, Stelmach & Tam, 1996, Zaroff, Knutelska, & Frumkes, 2003), there is evidence (Richards, 1970, 1971) that it could be as low as 80% with 20% stereoanomalous. Having made laboratory measurements of different aspects of stereo vision over the last 30 years, the first author has been surprised by how difficult it is to find subjects with otherwise normal vision who have good stereo sensitivity, unlike other 2-D visual functions, such as contrast sensitivity, color sensitivity, motion sensitivity, where this is not the case. Here, in two related studies (one in the general population and the other in a first-year university student class), we tested the stereo acuity of over 600 people using an online random dot stereo test in which the task was to discriminate (with unlimited viewing time) whether a square area defined by horizontal disparity alone was in front or behind the screen plane; one of the simplest tests of stereo function.

## Methods

### Stereo Depth Judgment Task

**Figure 1.**
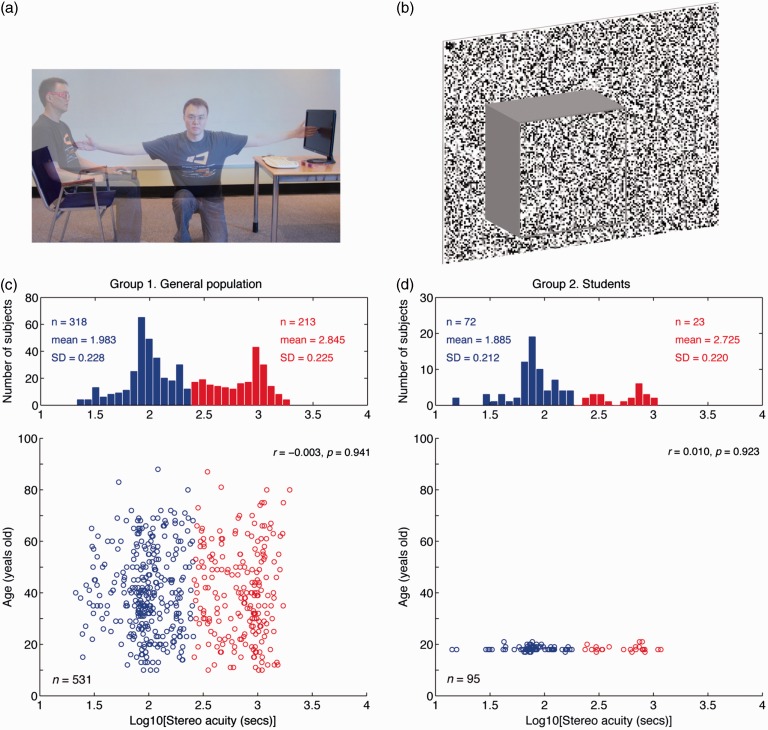
(a) Standardization for the testing distance for the web-based measurement. (b) Illustration of the random dot stimulus in which subjects have to detect a subfield perceived either in front of the screen or behind the screen. (c) Distribution of stereoacuities and their age dependence for the general population (*n* = 531). The results are well fitted by two different distributions, those with normal stereo acuity (blue) and those with poor stereo acuity (red). (d) Similar results for a student population (*n* = 95) but with a smaller percentage with poor stereopsis and a narrow age range.

The hybrid 2-AFC task involved, on each presentation, searching for the location (upper right, lower right, upper left, lower left) of a square box (see Figure 1(b)) that was defined only by disparity (and surrounded by zero disparity regions) and determining whether it was in front or behind the zero disparity fixation plane (monitor screen plane). There was no time limit, and the observer’s response which was indicated by a button press controlled when the next presentation occurred.

### Stimulus Generation

Each stimulus was a 1,024 × 768 pixel rectangle image made up of random noise patterns. The noise pattern comprised square dots. Each square dot was a three-by-three group of pixels. We generated a stereo subregion (394 × 384 pixels; width × height) using horizontal disparity. The screen resolution and color depth were obtained by Javascript running on the clients’ web browsers.

The subjects’ viewing distance varied from 40 cm to 200 cm as input by each viewer (see Figure 1(a)). In some cases, we used the height information input by each viewer to estimate the arm length span.

### Test Procedure

There were 11 disparity levels, ranging from 1 to 11 pixels. At the largest disparity of 11 pixels, the simulated depth was very obvious to stereo normal observers. We conducted two separate experiments.

#### Canadian broadcasting corporation experiment

The ﬁrst experiment was intended to be a rapid test suitable for screening a large subject sample with a simple stopping criterion to determine threshold. The stimuli were presented in increasing order of difficulty, starting from the easiest (i.e., largest disparity). To pass each level of difficulty, correct answers on two stimuli (of the same disparity) were required. At each presentation, a stimulus was drawn randomly from a set of eight pregenerated images (of the same disparity, where the box was in front or behind with equal probability at the four possible locations). The possible responses were “In Front,” “Behind,” and “Not Sure.” A test session terminated upon one of the following conditions:

- First incorrect answer
- Two consecutive “Not Sure” answers
- Correct answer at the most difficult stimulus.

The chromaticity of the stimuli was designed to provide dichoptic stimulation with amber-blue anaglyph glasses. The website can be accessed at 3d.mcgill.ca.

#### McGill psych100 experiment

A similar experiment was then repeated among the students attending Psych-100 class at McGill University with a narrow age range (around 20 years of age). We used a different set of anaglyph, red–green glasses, and hence, the chromaticity of stimuli was adjusted accordingly. Aside from this change, the order of stimulus presentation and stopping criterion remained the same.

### Data Analysis

To ensure valid entries, we filtered the entries using a number of criteria including the date of the entry (limited to one month), e-mail address (for the student group, only valid McGill addresses), IP address (limited to one for each subject), age (10–90 years), and indicated visual distance (>40 cm). This conservative approach greatly reduced our number of participants but increased the reliability of the web-based approach we used.

## Results

The results displayed in Figure 1(c, upper frame) are broadly distributed extending over a range of approximately 2.0 log units and can be modeled by two overlapping populations; one with a peak at 1.983 log arcsec (the *haves*) and another with a peak located at 2.845 log arcsec (the *have-nots*). There is no age dependence (lower frame).

A similar study within a student population (first-year psychology class at McGill University) produced similar results (Figure 1(d, upper frame)). Thus both groups exhibit a loss of stereo capabilities that extent to a large proportion of the population.

## Discussion

These results are surprising in that they suggest that there is a large percentage (32%) of people with below normal stereopsis. These stereo acuities vary over a 300:1 range with 24 to 40% of the population being 10 times worse. It is known that 5% of the population has amblyopia (lazy eye) and would be expected to have either poor or immeasurable stereo vision. It is hard to explain the current result in terms of subjects’ performing poorly due to lack of task comprehension because the student population exhibited a comparable dichotomous behavior. Another issue to consider is uncorrected refractive error as this would reduce stereo performance. However, this seems an unlikely explanation for two reasons. First, only a unilateral refractive error difference is likely to reduced stereo and to account for this degree of difference (approximately 1 log unit), it would need to be in excess of four dioptres, which is rare (incidence 1.9%; Mohammadi et al., 2013). Second, the students are less likely to have uncorrected refractive errors compared with the general population and yet they display comparable results. Oculomotor instability may also be a potential reason for the poor stereo performances as we assume subjects could adequately converge in the screen which represented zero disparity. Media opacities and age-related changes may also limit stereopsis if they are unilateral in the older age group, but the dichotomy reported here is not dependent on age.

We speculate that at the very least there is a large range of stereo capabilities in the normal population and that its likely cause is neural. It is likely that this is related to the original work of Richards (1970, 1971) who reported that about 20% of the population are stereo deficient, lacking either crossed or uncrossed disparity detectors. Our stimuli are very different from those used by Richards (1970, 1971), being global random dot stereograms, rather than local vertical lines, but like Richards (1970, 1971) our method also involves a signed disparity judgment. Studies that don’t use a signed disparity judgment, relying instead on the detection of depth or depth-defined shape, do not report high levels of stereo anomaly within the normal population (Birch et al., 2008). Nevertheless, it is surprising that stereopsis shows such individual variability when other visual sensitivities do not. The adult brain maintains a degree of neural plasticity, and it has recently been demonstrated that stereoscopic sensitivity in adults can be improved by training (Astle, McGraw, & Webb, 2011), so it remains a possibility that the *have-nots* can be transformed into *haves* with the right training.
